# Minimal Climate Impacts From Short‐Lived Climate Forcers Following Emission Reductions Related to the COVID‐19 Pandemic

**DOI:** 10.1029/2020GL090326

**Published:** 2020-10-22

**Authors:** James Weber, Youngsub M. Shin, John Staunton Sykes, Scott Archer‐Nicholls, N. Luke Abraham, Alex T. Archibald

**Affiliations:** ^1^ Centre for Atmospheric Science, Department of Chemistry University of Cambridge Cambridge UK; ^2^ National Centre for Atmospheric Science, Department of Chemistry University of Cambridge Cambridge UK

**Keywords:** aerosol, atmospheric chemistry, atmospheric composition, climate, climate change, COVID‐19

## Abstract

We present an assessment of the impacts on atmospheric composition and radiative forcing of short‐lived pollutants following a worldwide decrease in anthropogenic activity and emissions comparable to what has occurred in response to the COVID‐19 pandemic, using the global composition‐climate model United Kingdom Chemistry and Aerosols Model (UKCA). Emission changes reduce tropospheric hydroxyl radical and ozone burdens, increasing methane lifetime. Reduced SO_2_ emissions and oxidizing capacity lead to a decrease in sulfate aerosol and increase in aerosol size, with accompanying reductions to cloud droplet concentration. However, large reductions in black carbon emissions increase aerosol albedo. Overall, the changes in ozone and aerosol direct effects (neglecting aerosol‐cloud interactions which were statistically insignificant but whose response warrants future investigation) yield a radiative forcing of −33 to −78 mWm^−2^. Upon cessation of emission reductions, the short‐lived climate forcers rapidly return to pre‐COVID levels; meaning, these changes are unlikely to have lasting impacts on climate assuming emissions return to pre‐intervention levels.

## Key Points

- Emission reductions are likely to have led to a global reduction in short‐lived climate forcers and tropospheric oxidizing capacity
- Reductions in O_3_ and aerosol from both lower emissions and decreased sulfate oxidation resulted in a net negative radiative forcing
- The radiative impacts are small and short‐lived. Longer term climate impacts must come through future sustained emission reductions

## 1 Introduction

The outbreak of the COVID‐19 coronavirus disease in China in December 2019 and its global spread in early 2020 has led to the most deadly and disruptive pandemic in recent memory. As of 8 June, there have been 6.8 million confirmed cases and 395,000 deaths globally (World Health Organisation, 2020). In response, governments around the world have implemented varying lockdown measures. The resulting decreases in transport and economic activity have led to the unprecedented reduction of anthropogenic emissions of carbon dioxide (CO_2_) (Le Quéré et al., 2020) and short‐lived climate forcers (SLCFs) (Zhang et al., 2020). The SLCFs include sulfur dioxide (SO_2_), nitrogen oxides (NO and NO_2_, which together form NO_x_), carbon monoxide (CO), and organic carbon and black carbon (OC and BC, respectively). Such species perturb the oxidant balance of the atmosphere (O'Connor et al., 2020), the ozone budget (Young et al., 2018), and aerosol burden (Karset et al., 2018) and thus the radiative balance of the atmosphere and climate (Myhre et al., 2013). This paper aims to assess how the perturbations to atmospheric composition arising from changes to emissions of SLCFs due to the COVID‐19 pandemic affect parameters important for climate.

There remains uncertainty in the temporal, spatial, and composition changes to emissions arising from the restrictions imposed. Le Quéré et al. (2020) calculated reductions in daily CO_2_ emissions of between 11% and 25% by April 2020 relative to April 2019. Despite this uncertainty, there exist common themes to emissions changes on which this study focuses.

## 2 Methods

### 2.1 Model Description

Five experiments were performed using the United Kingdom Chemistry and Aerosols Model (UKCA) run at a horizontal resolution of 1.25° × 1.9° with 85 vertical levels up to 85 km (Walters et al., 2019) with the fully interactive stratospheric and tropospheric chemistry (Archibald et al., 2020) and GLOMAP‐mode aerosol scheme which simulates sulfate, sea‐salt, BC, organic matter, and dust but not currently nitrate aerosol (Mulcahy et al., 2020). Emissions of well‐mixed greenhouse gases (WMGHGs), such as methane (CH_4_) and CO_2_, were not simulated; rather, a prescribed value is applied for CO_2_ and a lower boundary condition used for methane, N_2_O and CFCs. The simulations were run using nudging (Telford et al., 2008) to atmospheric reanalyses from ECMWF (Dee et al., 2011) to constrain the simulations to consistent meteorology enabling a small ensemble of three different years: 2012, 2013, and 2014. The years chosen were the most recent where CMIP6 historical emissions were available and were averaged to filter out the influence of interannual meteorological variation. Nudging prevented temperatures and horizontal winds from responding to the forcings produced by the emissions changes, thus limiting the effect the changes in aerosols could have on clouds and the subsequent impacts on the radiation budget (Zhang et al., 2014).

### 2.2 Scenario Descriptions

Five scenarios were considered, each with different perturbations to emissions (Table 1). Emitted species are specified in Table S1. The perturbation scenarios A1–A4 were developed by reducing global anthropogenic emissions in the aviation, surface transport, and industrial sectors by a set factor. In all perturbation scenarios, emissions were held at the control run values until mid‐February before declining linearly until mid‐March to their minimum value. They remained at their minimum value until mid‐May before increasing linearly to the control levels by mid‐June (Figure S1). We made the approximation of all countries in the world making parallel emission reductions. As these scenarios were developed early in the COVID‐19 pandemic when information on the impact of the lockdown on all sectors was unknown, we drew on available information from a number of sources to compile emission reduction scenarios that span likely representative changes in emissions. See Text S1 for further details.

**Table 1:** Scenarios and Emission Changes

Scenario	Transport	Aircraft	Industry	% Global change in surface emissions during “lockdown period” (March–May)			
				NO	SO_2_	BC	OC
Control	No reduction	No reduction	No reduction	No reduction	No reduction	No reduction	No reduction
A1	−50%	−50%	−25%	−15.8	−8.84	−11.88	−3.66
A2	−50%	−25%	−25%	−15.8	−8.84	−11.88	−3.66
A3	−75%	−50%	−25%	−22.2	−9.48	−16.48	−4.52
A4	−50%	−50%	No reduction	−12.8	−1.27	−9.19	−1.73

The scenarios were designed to allow a comparison between the effects of decreasing different sectors on emissions. By comparing A1 with A3 and A4, we saw that global NO_x_ emissions were approximately twice as sensitive to surface transport emissions than industrial emissions, while the majority of SO_2_ emission decreases were due to industrial emissions. Comparing the primary aerosol emissions, BC was more sensitive to the surface transport sector, while OC was more sensitive to industry. While reducing aviation emissions resulted in a negligible decrease in the total mass of emissions, these emissions were injected directly into the free troposphere which is more sensitive to NO_x_ emissions (Stevenson et al., 2004). These reductions are in line with those in the recently published studies by Le Quéré et al. (2020) and Forster et al. (2020), which estimated decreases in aviation of 50–90%, surface transport of 40–75%, and various industrial emissions, such as Chinese coal (40%) and US steel (35%).

## 3 Results

In all cases, we combined the results from the simulations with different years of meteorology to generate an ensemble mean and compared the results of the different scenarios (A1–A4) to the control case. In all the scenarios, the effects of emission changes were short‐lived, and atmospheric composition returned to control levels within 2 months of the emissions reductions ceasing. In the following analyses, we focus on the peak lockdown period (mid‐March to mid‐May), where emissions are prescribed to be at their lowest, and quantify changes in composition and average Instantaneous Radiative Forcing (IRF) from ozone (O_3_) and aerosol direct effects.

### 3.1 Evaluation of NO_2_ Column

**Figure 1:**
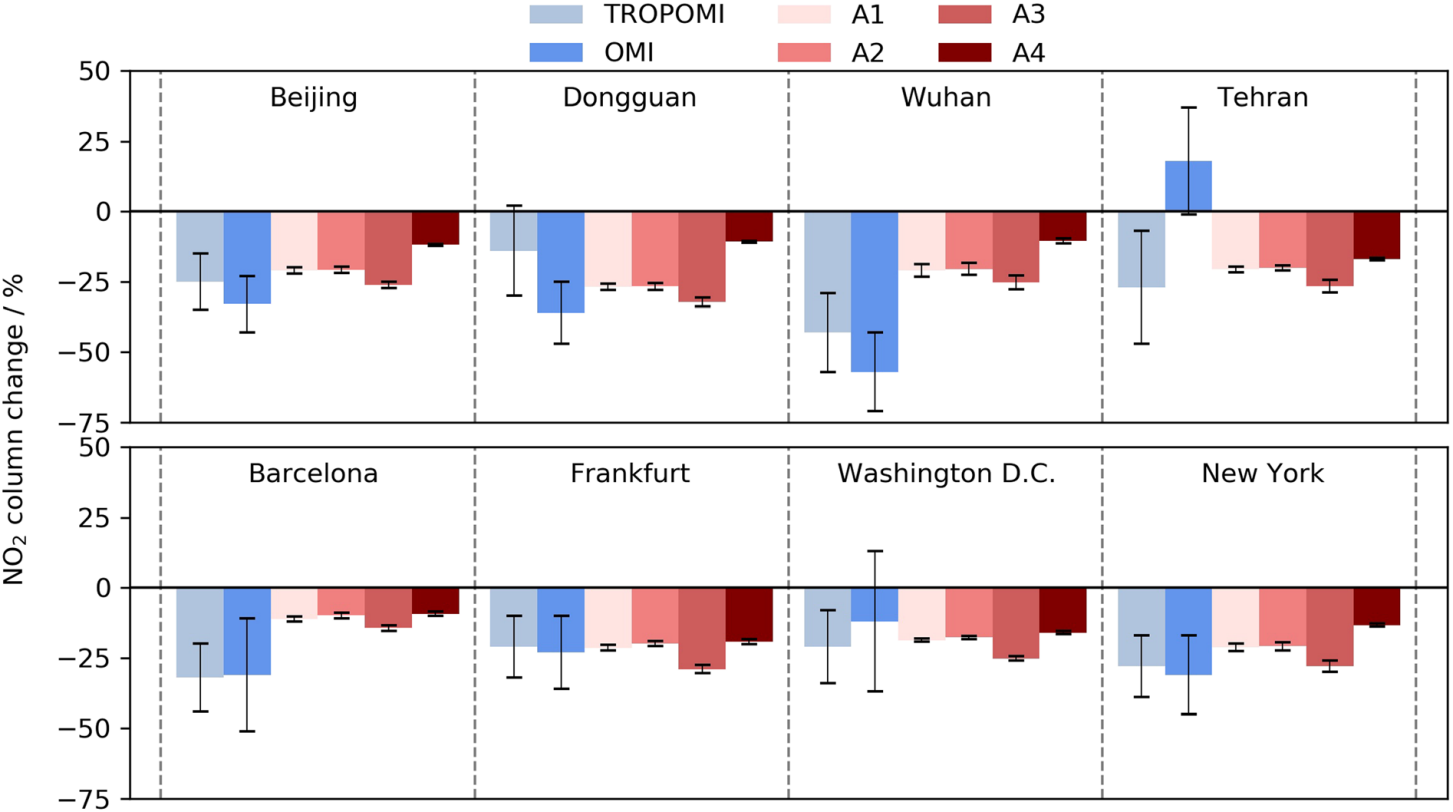
Observed and modeled tropospheric NO_2_ column changes. Observations are from TROPOMI and OMI relative to 2019; see Bauwens et al. (2020) for more details. Model results are from the four scenarios relative to the control averaged over the period of lowest emissions (mid‐March to mid‐May).

Observations of tropospheric NO_2_ columns have exhibited significant reductions globally (Bauwens et al., 2020; Zhang et al., 2020) with decreases in excess of 20% over many major cities. Figures 1 and S2 show NO_2_ column changes from observation (Bauwens et al., 2020) and model scenarios.

**Figure 2:**
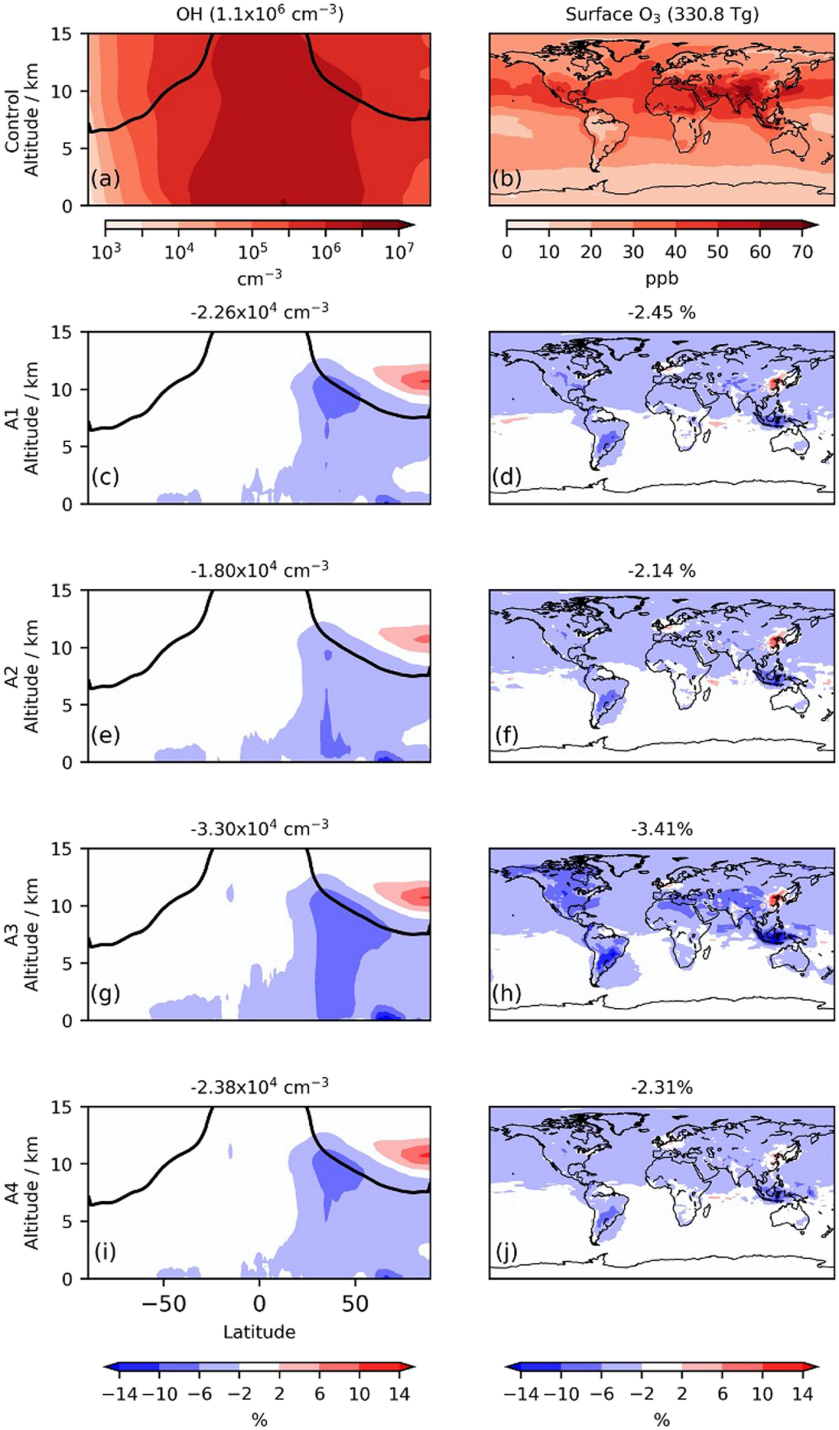
Zonal mean OH and surface O_3_ mixing ratios in control runs and respective changes (mid‐March to mid‐May). Model results are the ensemble mean for each scenario. Black lines in the OH plots show the tropopause. Titles in the left column show mean tropospheric air‐mass‐weighted (OH) in control (top) and change (lower panels). Titles in the right column show mean tropospheric O_3_ burden in control (top) and change (lower panels).

Figure 1 highlights that our model simulations are in good agreement with observed NO_2_ column decreases by Bauwens et al. (2020), with the A1 scenario being within error in most cases. This increases confidence in the representativeness of our emissions scenarios for the COVID‐19 changes. However, we note that the model simulations generally underestimate the magnitude of NO_2_ column changes, suggesting our emission perturbations may be at the lower end of what happened during the pandemic. Shi and Brasseur (2020) showed through surface observation analyses across China that the COVID‐19 lockdowns resulted in significant decreases in NO_2_, but increases in ozone (O_3_). These local increases in surface O_3_ in polluted regions are also captured in our simulations, although with a smaller magnitude (Figure 2), and are driven by the nonlinear NO_x_‐VOC chemistry that produces O_3_ in the troposphere (Monks et al., 2015). However, all scenarios exhibited a general decrease in global tropospheric O_3_, attributed to the reduction in NO_x_ emissions.

### 3.2 Reduction in Oxidant Burden

Globally averaged, the changes to emissions from transport, industry, and aviation led to decreases in the tropospheric O_3_ burden of 2.0–3.2% (Figures 2 and S3), which recovered quickly once emissions increased. The OH concentration was also simulated to have decreased (Figure 2). The reduction in tropospheric O_3_ was most pronounced in A3 where localized decreases exceeded 7%, illustrating the large impact of reducing surface transport emissions (Figure S4). The Northern Hemisphere midlatitudes, the location of the largest absolute change in emissions, saw the greatest reductions. Spatial heterogeneity in OH and O_3_ decrease between scenarios (Figure S5) revealed the importance of emissions from surface transport and aviation. The additional decreases in low‐altitude O_3_ and OH in Scenario A3 relative to A1 were attributed to the greater reduction in surface transport emissions in A3, while smaller decreases in midaltitude O_3_ and OH in A2 were due to the smaller reduction in aviation emissions. By comparing the oxidant distributions of A2–A4 with A1 (Figure S5), we can isolate the effects of different sectors. Aircraft NO_x_ emission reductions decreased O_3_ and OH predominantly in Northern Hemisphere mid‐upper latitudes around 10 km, while surface transport emission reductions reduced OH and O_3_ at lower altitudes around 30–50°N. The similarity in O_3_ and OH between scenarios A1 and A4 highlights the insensitivity of the tropospheric oxidant budget to industrial emissions.

The decrease in tropospheric OH did not affect model methane concentration due to the fixed methane surface boundary condition. However, the change in methane concentration, *c*, which would have occurred can be calculated from the methane lifetime (Equation 1) (Thornhill et al., 2020), where *f* = 1.33 is methane's feedback on its own lifetime (Fiore et al., 2009).

(1) $$\frac{\mathrm{\Delta c}}{c}={\left(\frac{\Delta \tau }{\tau }+1\right)}^{f}-1\approx f\frac{\Delta \tau }{\tau }$$

Methane lifetime increased by 2–2.5% (A1, A2, and A4) and 4% (A3) over the period of emissions reduction. Had steady state been reached, this would have corresponded to increases in methane concentration of ~20–40 ppb. However, since the duration of the perturbation was much shorter than the modeled lifetime of methane (~9.5 years), we determined a much smaller upper bound of 0.1% (2 ppb), using a simple kinetic model (Text S2). We therefore conclude the effect of oxidant changes on methane concentration and the associated forcing are negligible. This work does not account for changes in anthropogenic methane emissions, resulting from COVID‐19 lockdown measures, which are estimated to be smaller than 5% (Forster et al., 2020).

### 3.3 Reduction in Sulfate Aerosol Burden

**Figure 3:**
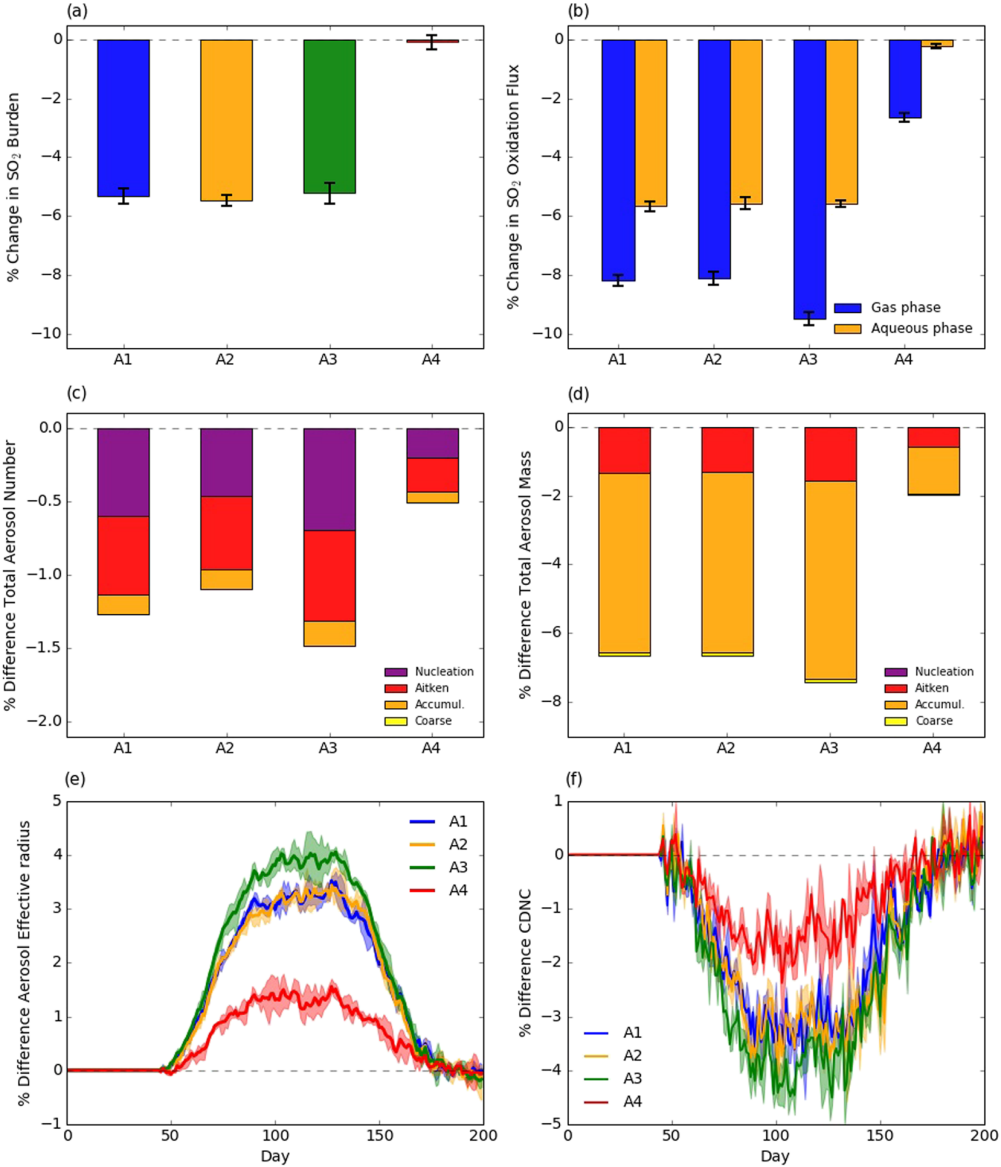
Mean change in (a) SO_2_ burden, (b) SO_2_ oxidation flux, (c) sulfate aerosol number, and (d) mass burden split by aerosol size (March–May). Mean change in (e) *r*_eff_ and (f) CDNC (error bars and shading show ensemble range).

The perturbation to oxidants reduced the oxidation flux of SO_2_ beyond the change due to the reduction in SO_2_ emissions alone, illustrating the coupling between emissions, oxidants, and sulfate aerosol, an important climatic forcer. SO_2_ production fluxes (emissions plus chemical production) decreased by around 8% (A1–A3) and 1.3% (A4), highlighting the sensitivity of SO_2_ to industrial emission reductions. However, the corresponding drop in SO_2_ burden (5.4% [A1–A3) and 0.1% [A4]) (Figure 3a) was smaller than the production flux decrease due to a reduction in chemical loss driven by oxidant decreases. This effect was most pronounced with the tropospheric gas phase OH + SO_2_ flux which decreased by 8–9.5% (A1–A3) and 2.6% (A4) (Figure 3b) and showed significant spatial similarity to (OH) change and exceeded the changes in SO_2_ alone (Figure S6).

The other SO_2_ oxidation pathway, aqueous oxidation by H_2_O_2_ and O_3_, decreased by only 4% (A1–A3); meaning, relatively more SO_2_ was oxidized via aqueous phase chemistry. This is important because in UKCA, the H_2_SO_4_ produced via OH + SO_2_ oxidation can nucleate new particles and thus affects aerosol number and size distribution. However, the aqueous phase pathway only adds mass to existing particles. The different reductions in gaseous and aqueous flux cause an additional perturbation to the aerosol size distribution resulting in fewer, larger aerosols (Figures 3c and 3e).

We calculated a reduction in sulfate aerosol burden (with rapid post‐lockdown recovery) with nonuniform reduction across the aerosol modes and largest changes in the midlatitude Northern Hemisphere (Figure S7). The largest decrease in mass occurred in the accumulation mode (Figure 3c) and the largest decrease in number in the nucleation mode (Figure 3d). This perturbation to the size distribution produced an increase in the mean aerosol effective radius (*r*_eff_) of 1–4% (Figure 3e) and is attributed in part to the greater relative reduction of gas phase oxidation of SO_2_ (and thus new particle nucleation) than aqueous phase oxidation: a further illustration of coupling between composition and climatically relevant agents.

The perturbation to the aerosol size and number distribution resulted in cloud droplet number concentration (CDNC) decreases of up to 4% globally (Figure 3f), with localized decreases exceeding 10% (Figure S8) and commensurate increases in effective cloud droplet radius of 0.25–0.4%.

### 3.4 Aerosol Optical Depth

The decrease in simulated sulfate aerosol burden and emissions of primary aerosol (BC, OC) resulted in decreases in aerosol optical depth (AOD) at 550 nm across most terrestrial regions in scenarios A1–A3 (Figure S9) with rapid recovery as emissions returned to pre‐lockdown levels. Eastern China exhibited the largest absolute decreases, while A4 showed much smaller decreases, highlighting the major contribution of industrial SO_2_ emissions to AOD. Observed AOD changes between 2017–2019 and 2020 from VIIRS (Sayer et al., 2018) were analyzed (Figure S10) but showed little significant signal due to considerable noise and complex regional effects.

## 4 Radiative Effects

Radiative forcings were calculated as the difference in Top Of Atmosphere (TOA) outgoing flux between the perturbed and control runs over the 3‐month emissions reduction period, averaged over 3 years and decomposed into aerosol direct radiative effects (*IRF*_DRE_) (Equation 2), aerosol‐cloud effects (*CRE*) (Equation 3), and clear‐sky effects (*CS*) (Equation 4) following the equations from Ghan (2013):

(2) $$\mathrm{IR}F_{\mathrm{DRE}}=\Delta \left(F-F_{\text{clean}}\right)$$

(3) $$\mathrm{CRE}=\Delta \left(F_{\text{clean}}-F_{\text{clear},\text{clean}}\right)$$

(4) $$\mathrm{CS}=\Delta \left(F_{\text{clear},\text{clean}}\right)$$

where *F* is the TOA radiative flux, *F*_clean_ the flux excluding scattering and absorption by aerosols, and *F*_clear,clean_ the flux excluding scattering and absorption by aerosols and clouds.

**Figure 4:**
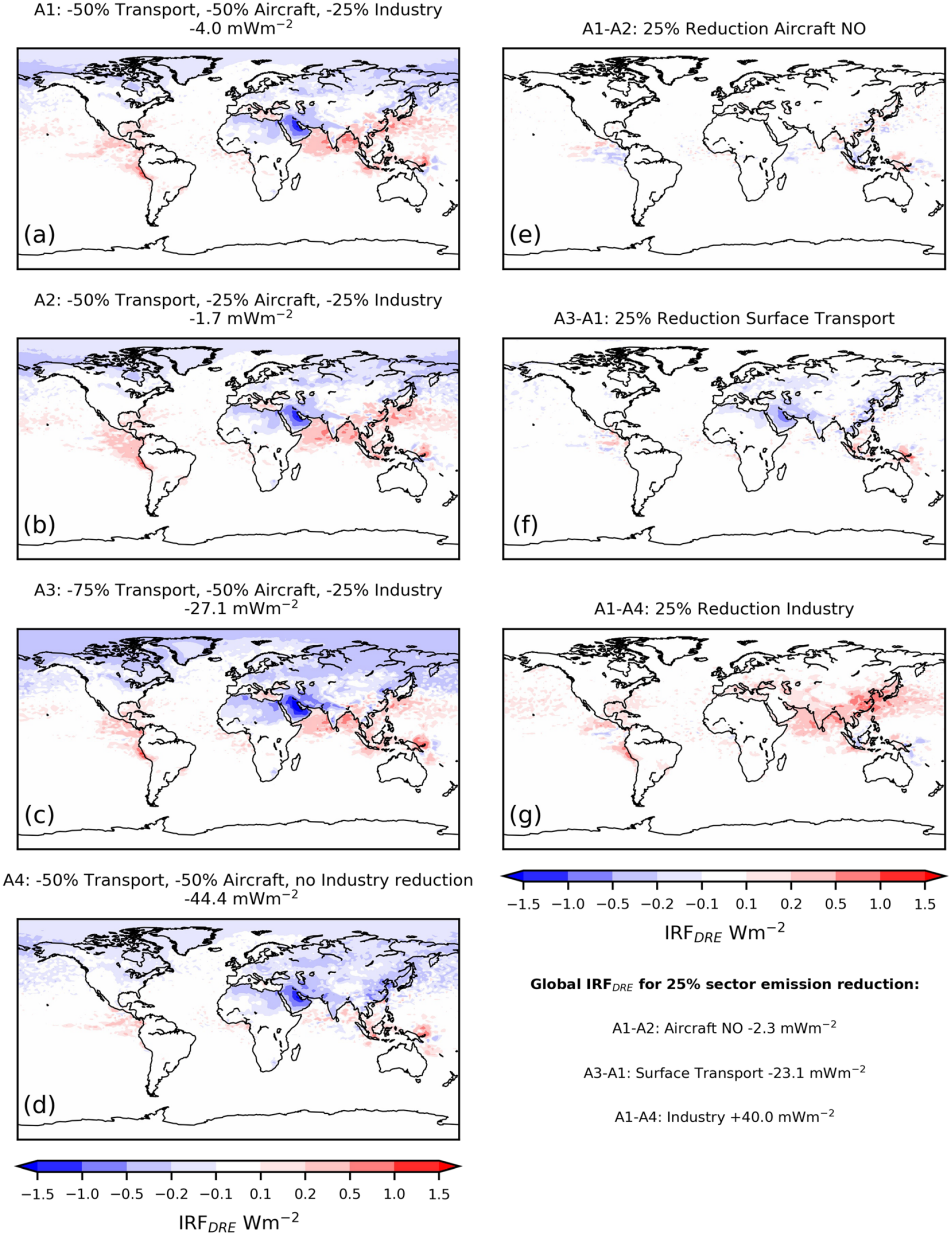
IRF from aerosol direct effects (*IRF*_DRE_) for (a–d) all perturbed scenarios relative to control and between perturbed scenarios isolating the *IRF*_DRE_ sensitivity to 25% reductions in (e) aircraft NO emissions, (f) surface transport emissions, and (g) industrial emissions. *IRF*_DRE_ shown for mid‐March to mid‐May, averaged over 3 years, values above figures (a)–(d) show area‐weighted mean over period.

The net radiative forcing was small and determined to be statistically insignificant at the 95% confidence interval for all scenarios over most of the globe, with the only statistically significant region being the Arabian Peninsula (Figure S11). This was attributed in part to the offsetting effects of BC and ozone reduction and sulfate reduction (Table 2; Figure 4). The drop in CDNC from aerosol reduction was expected to reduce cloud albedo (Twomey, 1977), but the radiative forcing from aerosol‐cloud interactions was not found to be statistically significant in this study (95% confidence) (Figure S12), at the global scale. This insignificance was attributed to the high variability in clouds which resulted in interannual differences in the sign of global radiative forcing from aerosol‐cloud interactions. Despite its statistical insignificance, the range of aerosol‐cloud forcing values (−57 to +96 mWm^−2^) was larger in magnitude than the forcings from O_3_ and the aerosol DRE (Table 2). While the variability means it was not possible to determine a value for the aerosol‐cloud forcing with confidence, future work to discern a statistically significant signal (employing many more ensemble members) is a priority since the possible size of the signal could be large enough to qualitatively change our conclusions on the climatic impact of the COVID‐19 emission changes.

**Table 2:** RF Relative to Control Runs Averaged Over the Period of Lowest Emissions (Mid‐March to Mid‐May)

Radiative forcing/mWm^−2^	A1	A2	A3	A4
Ozone (adjusted RF)	−37 (−38 to −35)	−31 (−32 to −31)	−51 (−52 to −49)	−35 (−36 to −35)
Aerosol direct effect IRF	−4 (−9 to +3)	−2 (−8 to +6)	−27 (−34 to −18)	−44 (−47 to −40)
Ozone and aerosol RF	−41	−33	−78	−69

*Note*. Values in parentheses show the ensemble range.

The clear sky component of the radiative forcing was also determined to be statistically insignificant across the globe (Figure S13), and this was attributed to the large variability in forcing agents such as water vapor, which yielded a low signal‐to‐noise ratio and necessitated the use of offline methods to determine forcing from the ozone column change. The impact of ozone column reduction (Figure S14) on radiative forcing was estimated using the conversion factor of 0.042 Wm^−2^ DU^−1^ based on the calculations of Stevenson et al. (2013) of stratospherically adjusted RFs from changes in tropospheric ozone. Changes to ozone were mostly located in the troposphere, but small effects were also seen in the lower stratosphere (Figure S4).

The *IRF*_DRE_ was calculated to be substantially smaller than the O_3_ forcings in A1 and A2 but comparable in A3 and A4. Despite the warming effect expected from the reduction in sulfate aerosol, the global aerosol IRF was simulated to be negative in all scenarios (Table 2).

Spatial analysis of the *IRF*_DRE_ (Figure 4) revealed a negative forcing over large Northern Hemisphere terrestrial regions except Eastern China which exhibited a warming in A1–A3. Calculating the *IRF*_DRE_ between perturbed scenarios (Figures 4e–4g) allowed the sensitivity to emission perturbations by sector to be determined. The 25% reduction in industrial emissions resulted in a globally averaged positive forcing of 40 mWm^−2^. The *IRF*_DRE_ in areas of China, where the reduction in SO_2_ emissions and sulfate column was greatest (Figure S15), exceeded 1 Wm^−2^, illustrating the local and global climatic impacts that changes in important aerosol precursors can have.

By contrast, reducing surface transport led to a negative forcing of −23 mWm^−2^ with the strongest cooling over the Arabian Peninsula, which can be seen in the *IRF*_DRE_ of all scenarios when compared to the control (Figures 4a–4d). This was attributed to the fact that the reduction in aerosol exposed solar radiation to a surface with a higher albedo than the original aerosol population, resulting in a greater fraction of insolation being reflected (Haywood & Shine, 1995). This effect was compounded over the Arabian Peninsula by the large decreases in BC emissions from both surface transport and industry sectors resulting in decreases of 20–40% to the BC column (Figure S16). BC is a strongly absorbing aerosol component with low single‐scattering albedo (Bond et al., 2013), and accordingly, the increase in single‐scattering albedo (Figure S16) is most pronounced over the Arabian Peninsula and correlates well with the negative *IRF*_DRE_. In addition, the positive forcing from industrial emissions reduction was much more modest in this region (Figure 4g), and therefore, the associated warming effects were smaller. Globally, these competing aerosol forcing effects, stemming from BC emissions from surface transport and SO_2_ emissions from industry, almost completely offset in A1 and A2, while the greater reductions in BC from surface transport (75%) resulted in net cooling in A3 from the aerosol direct effect. The even larger cooling in A4 was attributed to the combination of BC emissions reduction from transport without the large reduction in SO_2_ emissions and aerosol column from industrial emissions, resulting in a higher SSA (Figure S16).

## 5 Conclusion

In this study, we investigated how global reductions in anthropogenic road transport, aviation, and industrial emissions comparable to those resulting from the international response to the COVID‐19 pandemic impacted atmospheric composition and radiative forcing due to SLCFs. Our model results have shown these emission reductions led to significant changes in atmospheric composition, driven by the changes in the oxidizing capacity of the troposphere and oxidant‐aerosol‐precursor interactions. Decreases in NO_x_ emissions reduced tropospheric O_3_ and, as a result, the oxidizing capacity, with concomitant increases in methane lifetime—although because of the long lifetime of methane relative to the duration of the emission perturbations a negligible increase in methane forcing. SO_2_ emission reductions and the reduction in tropospheric oxidizing capacity led to decreases in sulfate burden. The reduction in sulfate aerosol number is predominantly manifest in the nucleation mode and attributed in part to the greater relative reduction in gas phase SO_2_ oxidation compared to aqueous phase oxidation and supported by increases in aerosol effective radius and decreases in CDNC. This highlights the influence of oxidant changes on the aerosol size distribution (as well as aerosol burden), an important climatic parameter.

SO_2_‐only emissions reductions similar in magnitude to our simulations have been shown to produce noticeable regional and global climatic effects in previous studies (Conley et al., 2018; Westervelt et al., 2017). Indeed, when comparing scenarios A1 and A4, where the largest difference is in SO_2_ emissions (due to a 25% reduction in industrial emissions), our model shows a positive forcing from the aerosol direct effect over China of a similar magnitude to Conley et al. (2018). Conversely, a 25% reduction in surface transport emissions (A1–A3) yielded a global negative radiative forcing from the aerosol direct effect, largely due to reductions in BC, with localized forcings exceeding −1 Wm^−2^ in the Arabian Peninsula. These effects largely offset each other and, when combined with the negative forcing from tropospheric O_3_ reduction, led to a small net forcing of −33 to −78 mWm^−2^. This change is short‐lived and comparable to a *temporary* and hypothetical decrease of 3–6 ppm of CO_2_.

The forcing from the clear sky and the aerosol‐cloud interactions components are statistically insignificant (at the 95% confidence level) due to the high variability of the forcing agents (particularly clouds and water vapor), which leads to large variation in the magnitude and sign of the response. This does not necessarily mean these responses to the COVID‐19 emissions reductions are not important (indeed, it is possible for the forcing for the aerosol‐cloud interactions to be larger in magnitude than the forcings from O_3_ and the aerosol DRE), rather that the signal is small compared to the noise over the relatively short timescale of the perturbation. While beyond the scope of this work, this finding is important for future investigations into the climatic response to COVID‐19 emission changes as they will require many ensemble members or long integrations (e.g., Conley et al., 2018) to determine statistically significant signals in the clear sky and aerosol‐cloud interactions contributions to the radiative forcing.

Our results suggest that temporary changes to SLCF emissions due to the COVID‐19 emergency measures are not going to have a significant impact on near‐term climate change. Our results reinforce the results of Forster et al. (2020), who estimate a cooling of 0.01°C as a direct result of the COVID‐19 lockdown. However, our use of UKCA, a state‐of‐the‐science 3D process‐based model, as opposed to the simple FaIR v1.5 model used by Forster et al. (2020), enables us to assess the regional and seasonal variations in the response and explicitly simulate the nonlinear combined effect of all SLCFs. While our work gives evidence for minimal climate impacts of the lockdown, a focus on green investment in economic recovery packages could have much more noticeable climatic effects (Forster et al., 2020). Elucidating the full effective radiative forcing and the climate response due to emission changes, including from aerosol‐cloud interactions, warrants further investigation using longer free‐running simulations.

## Conflict of Interest

All authors declare that they have no conflict of interests.

## Supporting information

Supporting Information S1

## Data Availability

All necessary data are available on the CEDA archive (http://data.ceda.ac.uk/badc/deposited2020/COVID19_emiss_reduc_study/data), and further information about the data can be found online (https://catalogue.ceda.ac.uk/uuid/b5ea7341a7164525b74143d8afe77223).
