# Supplementary material for: Minimal Climate Impacts From Short‐Lived Climate Forcers Following Emission Reductions Related to the COVID‐19 Pandemic
Source: Geophys Res Lett. 2020 Oct 22;47(20):e2020GL090326. doi: 10.1029/2020GL090326 (PMC7646061; doi:10.1029/2020GL090326)
Supplement: Supplementary file 1 — Supporting Information S1 [file GRL-47-e2020GL090326-s001.pdf]

Geophysical Research Letters

Supporting Information for

Minimal climate impacts from short-lived climate forcers following emission reductions related to the COVID-19 pandemic

James Weber[1], Youngsub M. Shin[1], John Staunton Sykes[1], Scott Archer-Nicholls[1], Nathan Luke Abraham[1,2] and Alex T. Archibald[1,2]

[1]Centre for Atmospheric Science, Department of Chemistry, University of Cambridge, Cambridge, CB2 1EW, UK
[2]National Centre for Atmospheric Science, Department of Chemistry, University of Cambridge, Cambridge, CB2 1EW, UK

Contents of this file

Figures S1 to S17
Text S1 to S2
Table S1

Introduction

The following is supporting information for the main text. It consists of additional plots of parameters calculated from processing UKCA output data, additional emission reduction information, further detail as to the calculation of methane concentration perturbation and additional information about the species emitted in the UKCA model.

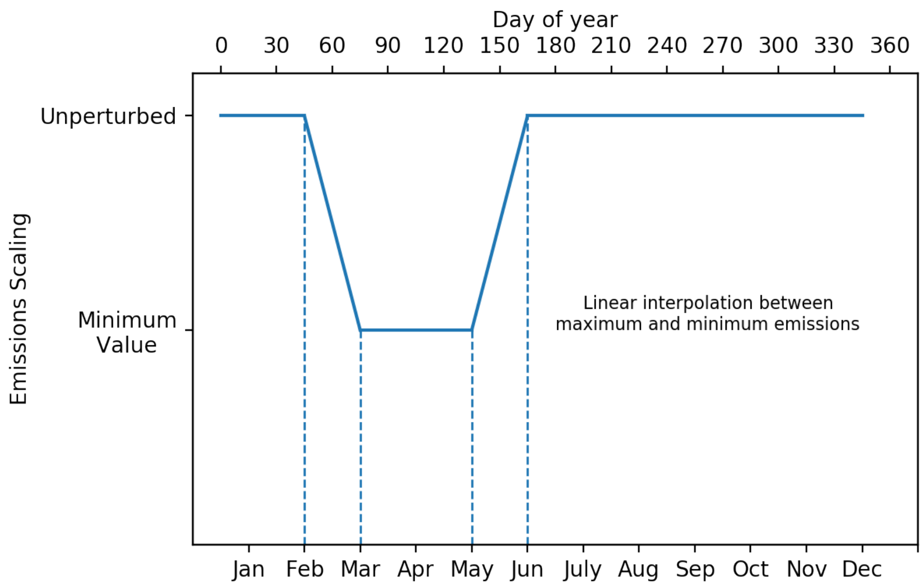

Figure S1. Emissions Time Series. Month ticks aligned to the middle of each month. Emitted values are updated every 5 days.

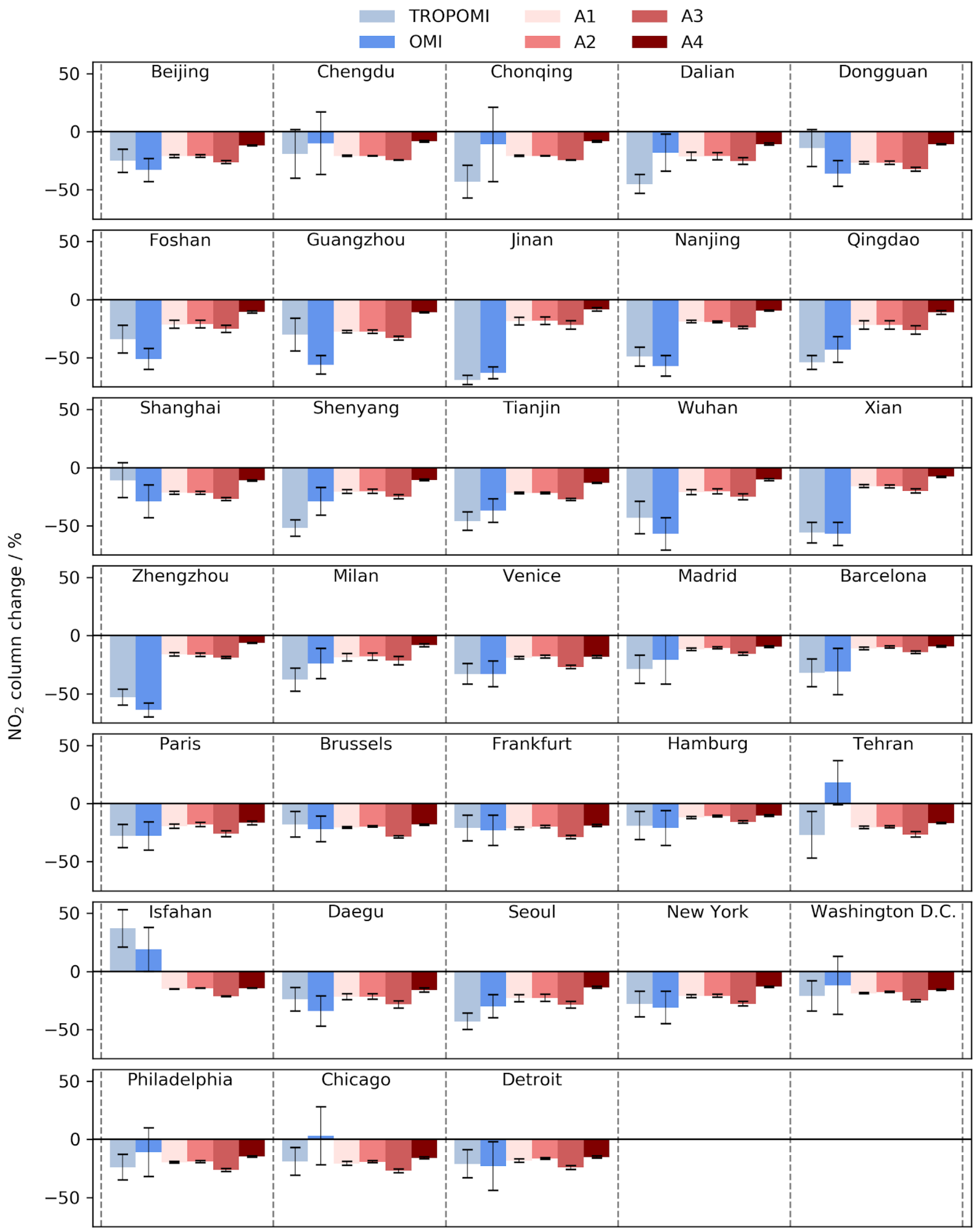

Figure S2. Observed and modelled tropospheric NO_2 column percentage change. Observations are from TROPOMI and OMI for the lockdown period (Feb to Mar) relative to 2019 (Bauwens et al., 2020) and model results from the 4 model scenarios relative to the control averaged over the period of emissions reduction (mid-Mar to mid-May)

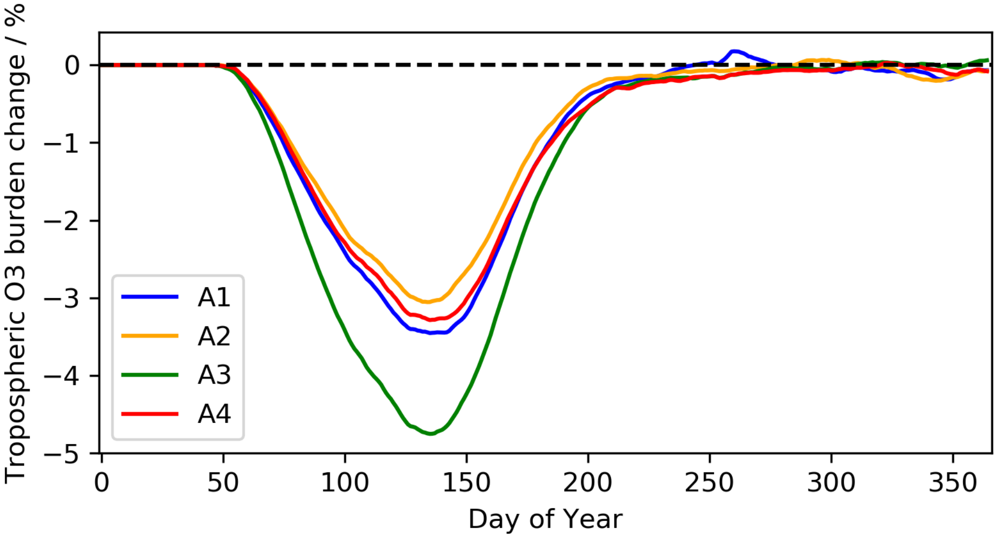

Figure S3. Modelled ensemble mean tropospheric ozone burden change compared to control. Tropopause diagnosed in-model using the WMO thermal lapse-rate tropopause definition (lapse-rate < 2℃ km^{-1}).

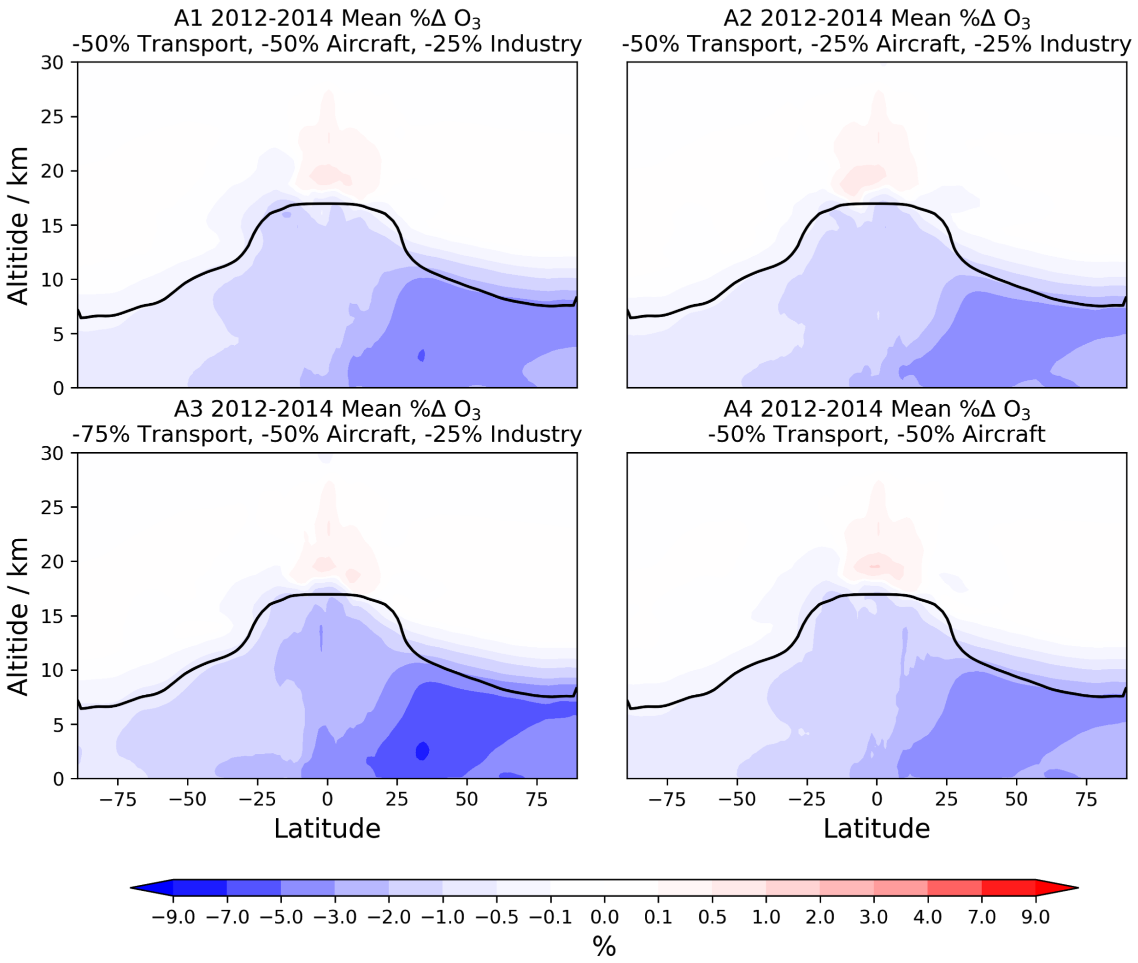

Figure S4. Zonal mean of percentage change in O3 mixing ratio (mid March - mid May) averaged over 3 years 2012-2014. Black lines show the tropopause.

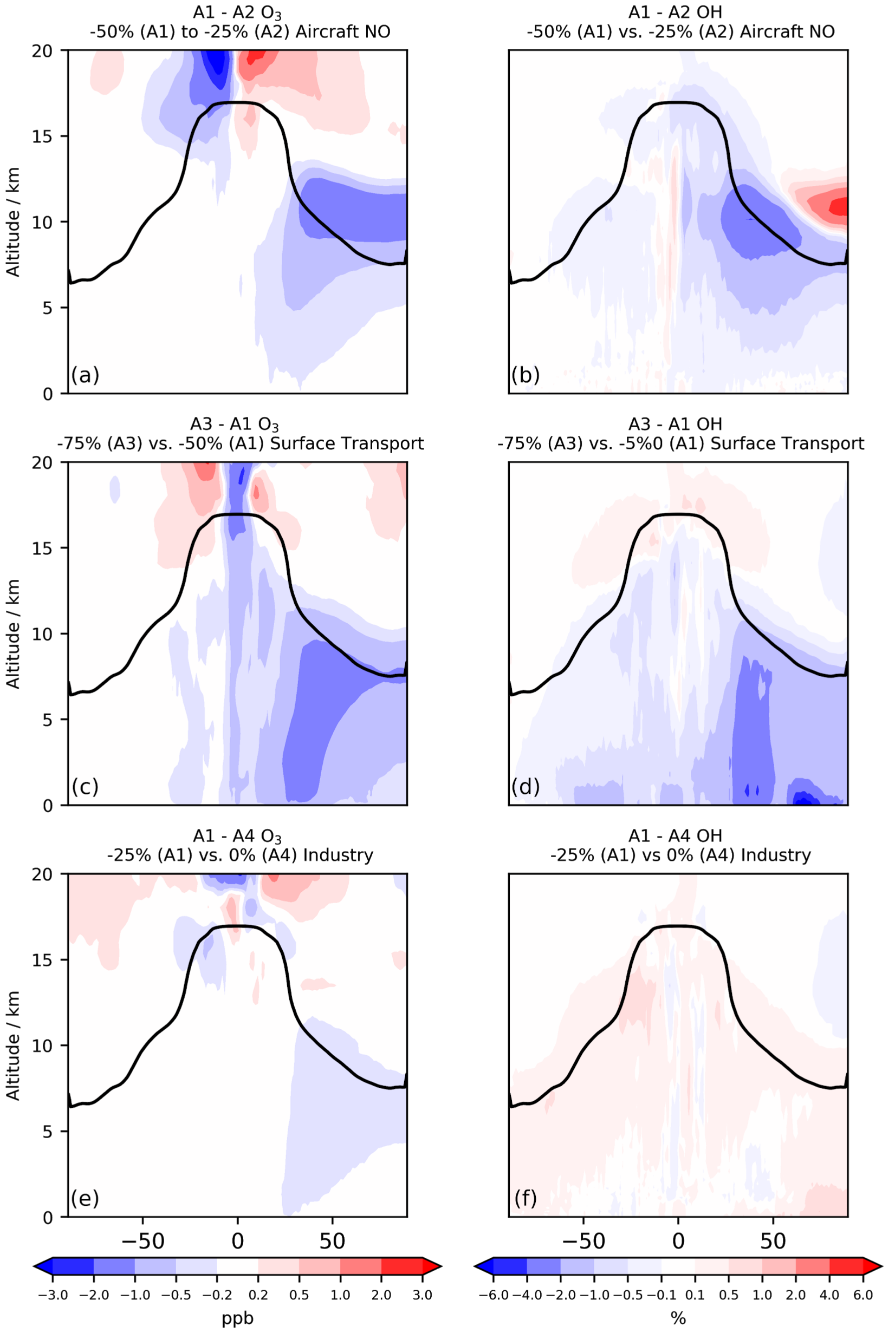

Figure S5. Zonal mean change in O_3 mixing ratio and zonal mean percentage change in OH mixing ratio between. In each case, the plot show the effect of reducing the emissions of the sector of interest (e.g. A1-A2 shows impact of reducing aircraft NOx emissions from a 25% reduction to a 50% reduction). All changes for mid March - mid May averaged over 3 years 2012-2014 Black lines show the tropopause.

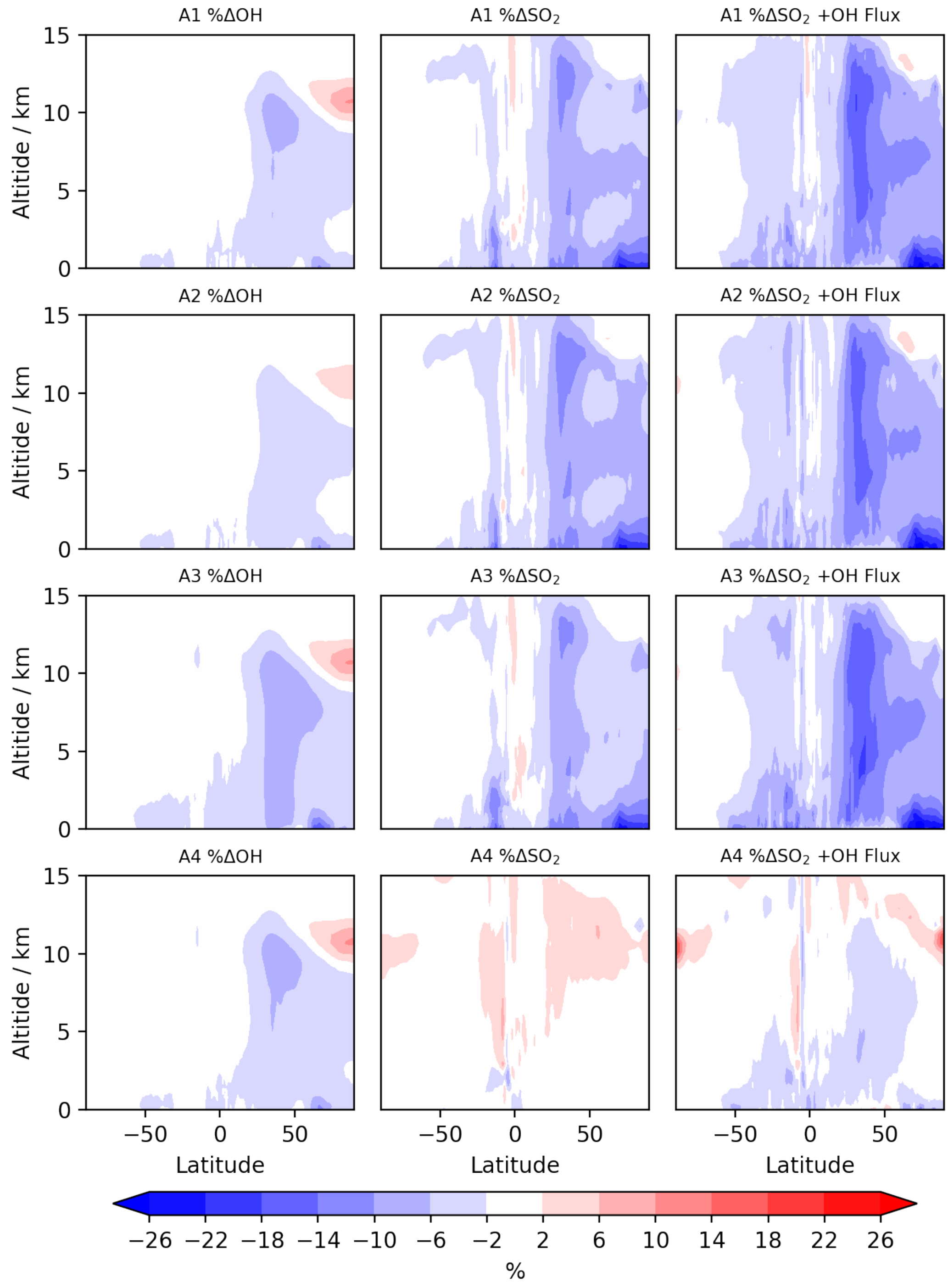

Figure S6. Zonal mean of percentage change in OH and SO_2 mixing ratios and SO_2 + OH flux (mid March - mid May) averaged over 3 years 2012-2014. The NH midlatitude spatial distribution of the flux change closely resembles changes in the OH field. Furthermore, there are regions where

the percentage change in the flux is larger than the percentage change in SO_2, illustrating the importance of the OH field perturbation.

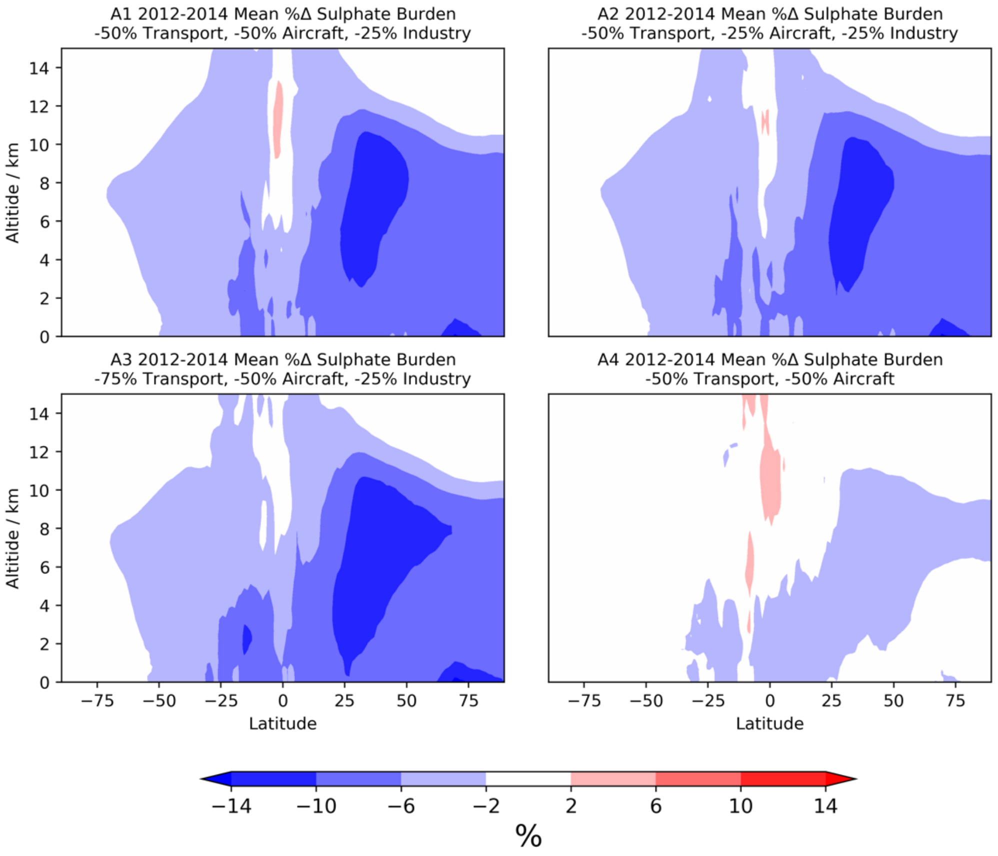

Figure S7. Zonal mean of percentage change in sulphate aerosol burden (mid March - mid May) averaged over 3 years 2012-2014.

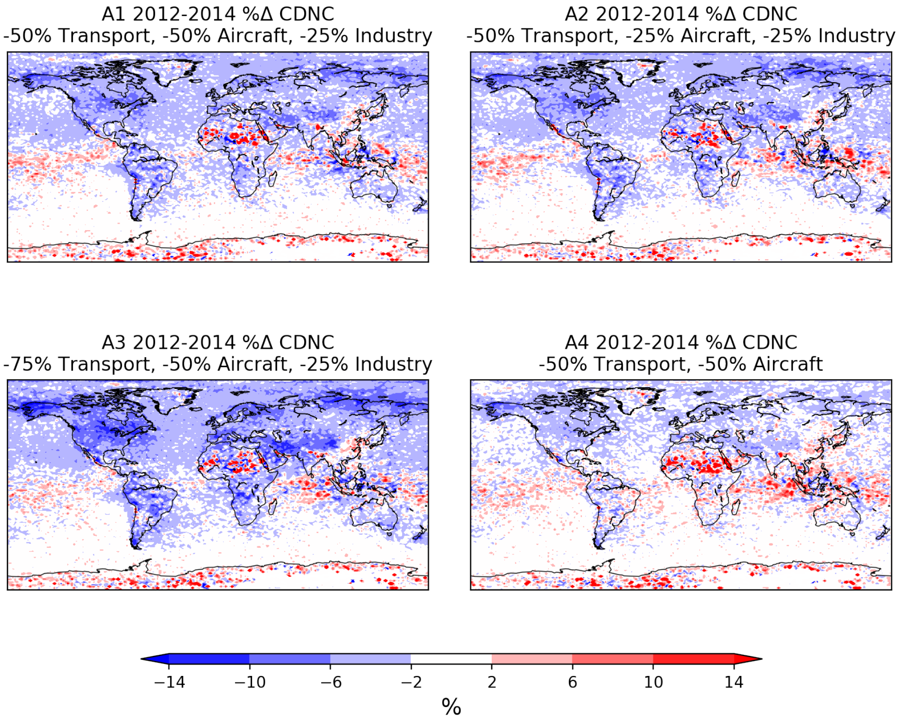

Figure S8. Percentage change in CDNC for (mid March - mid May) averaged over 3 years 2012-2014.

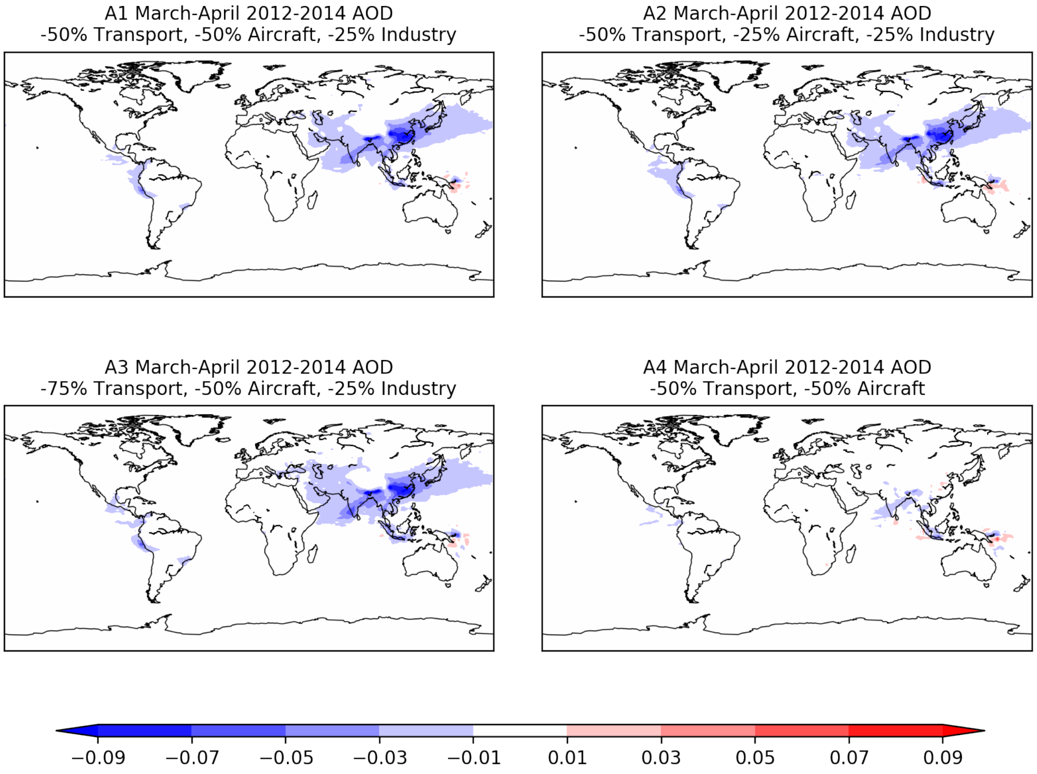

Figure S9. Changes in simulated AOD at 550 nm March-April between perturbed runs and control averaged over 3 years 2012-2014.

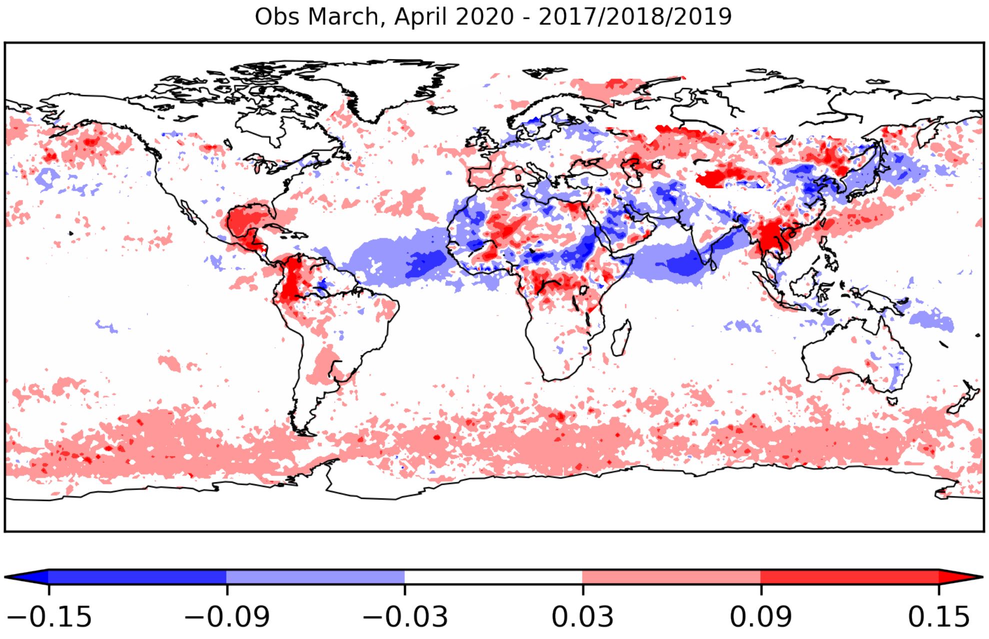

Figure S10. Changes in observed AOD 550 nm from VIIRS (Sayer et al., 2018) between March-April 2020 and mean of March-April 2017-2019.

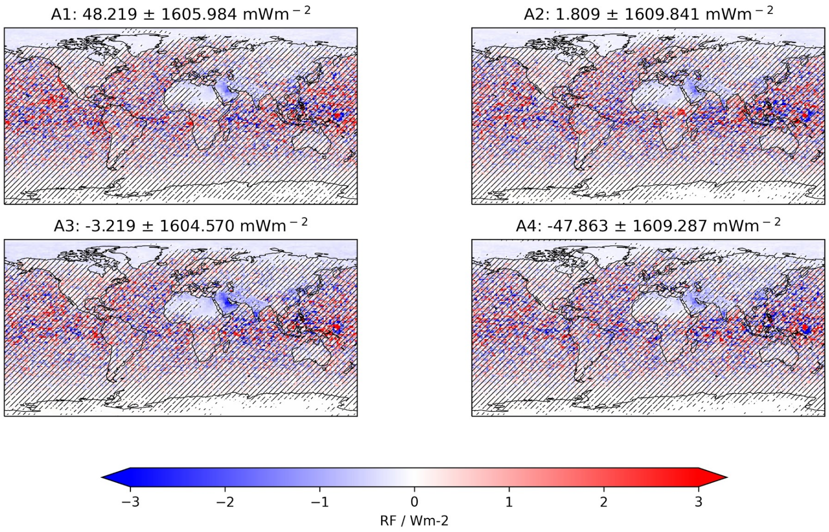

Figure S11. Total radiative forcing for the period of lowest emissions, averaged over 3 years. Hatching indicates regions where the radiative forcing is not statistically significant (95% confidence level). Values above each plot show the area-weighted mean with the mean 95% confidence interval.

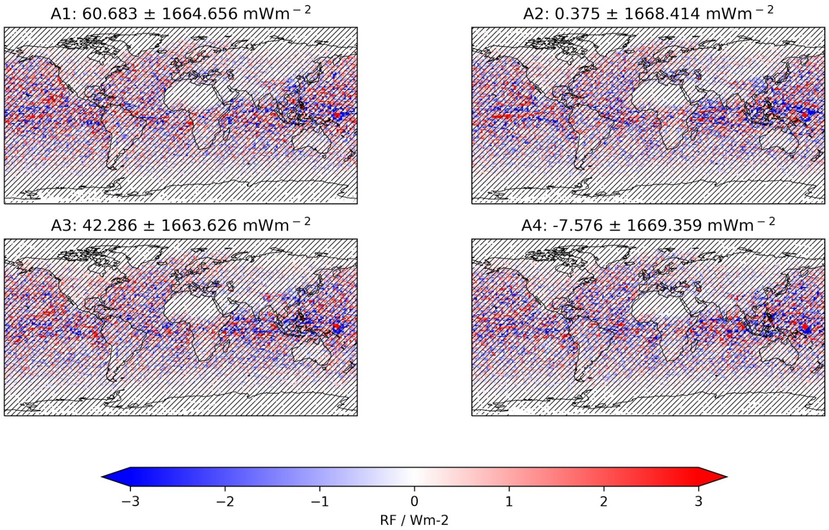

Figure S12. Radiative forcing from aerosol-cloud interactions for the period of lowest emissions, averaged over 3 years. Hatching indicates regions where the radiative forcing is not statistically significant (95% confidence level). Values above each plot show the area-weighted mean with the mean 95% confidence interval.

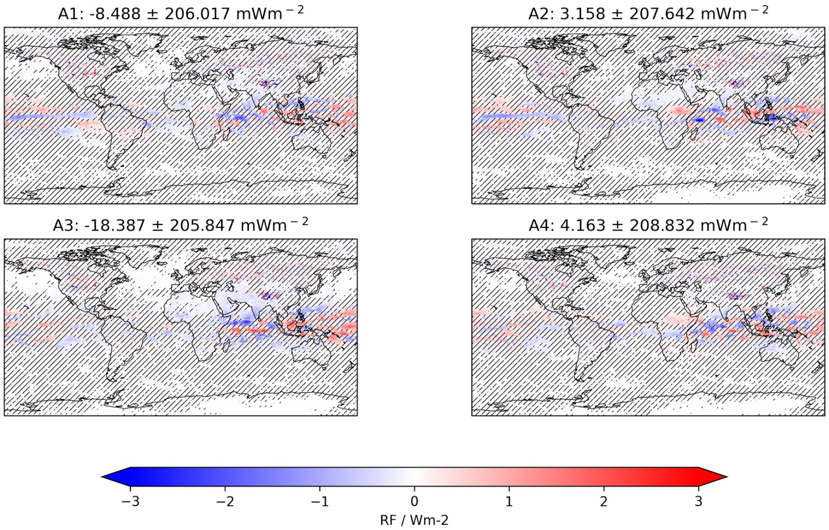

Figure S13. Clear sky radiative forcing for the period of lowest emissions, averaged over 3 years. Hatching indicates regions where the radiative forcing is not statistically significant (95% confidence level). Values above each plot show the area-weighted mean with the mean 95% confidence interval.

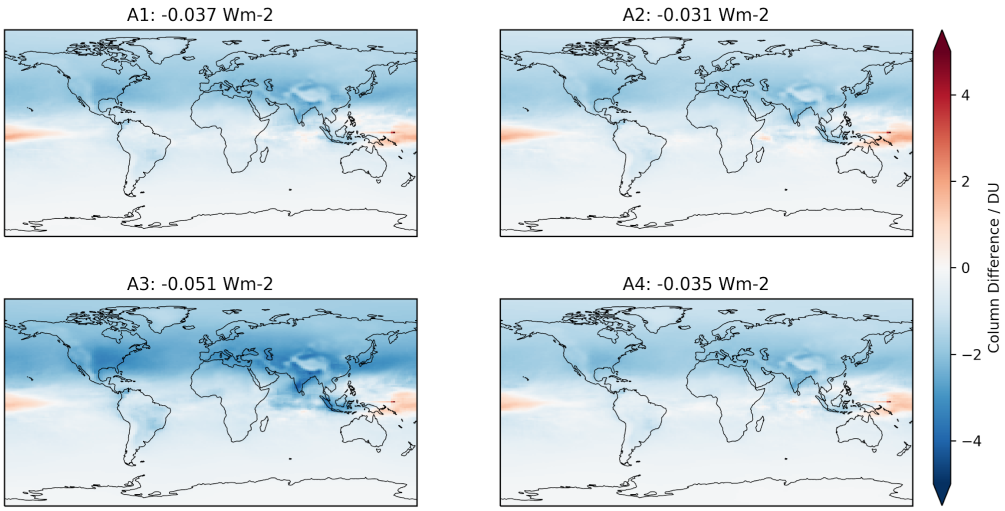

Figure S14. Percentage change in tropospheric O_3 column (mid March - mid May) averaged over 3 years 2012-2014 and instantaneous radiative forcing relative to control calculated using method from Stevenson et al (2013).

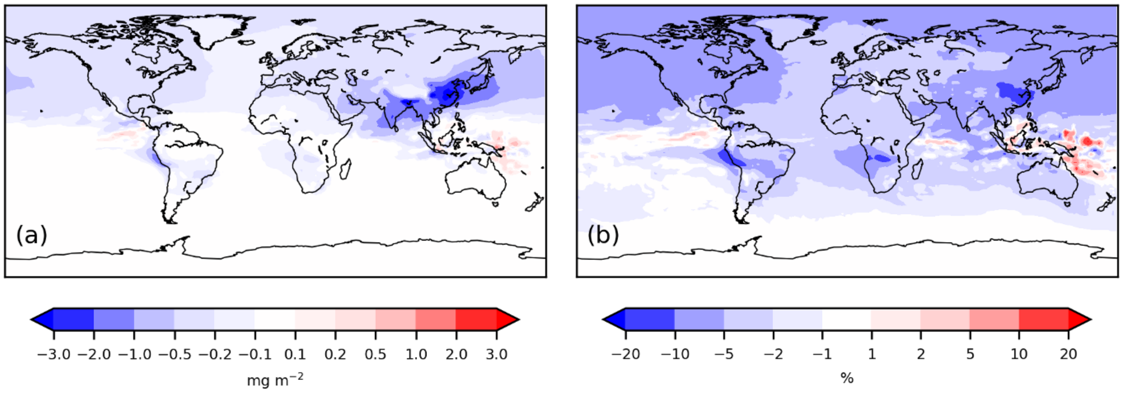

Figure S15. Absolute (a) and percentage change (b) in sulphate aerosol column (A1- A4) for mid March - mid May averaged over 3 years 2012-2014.

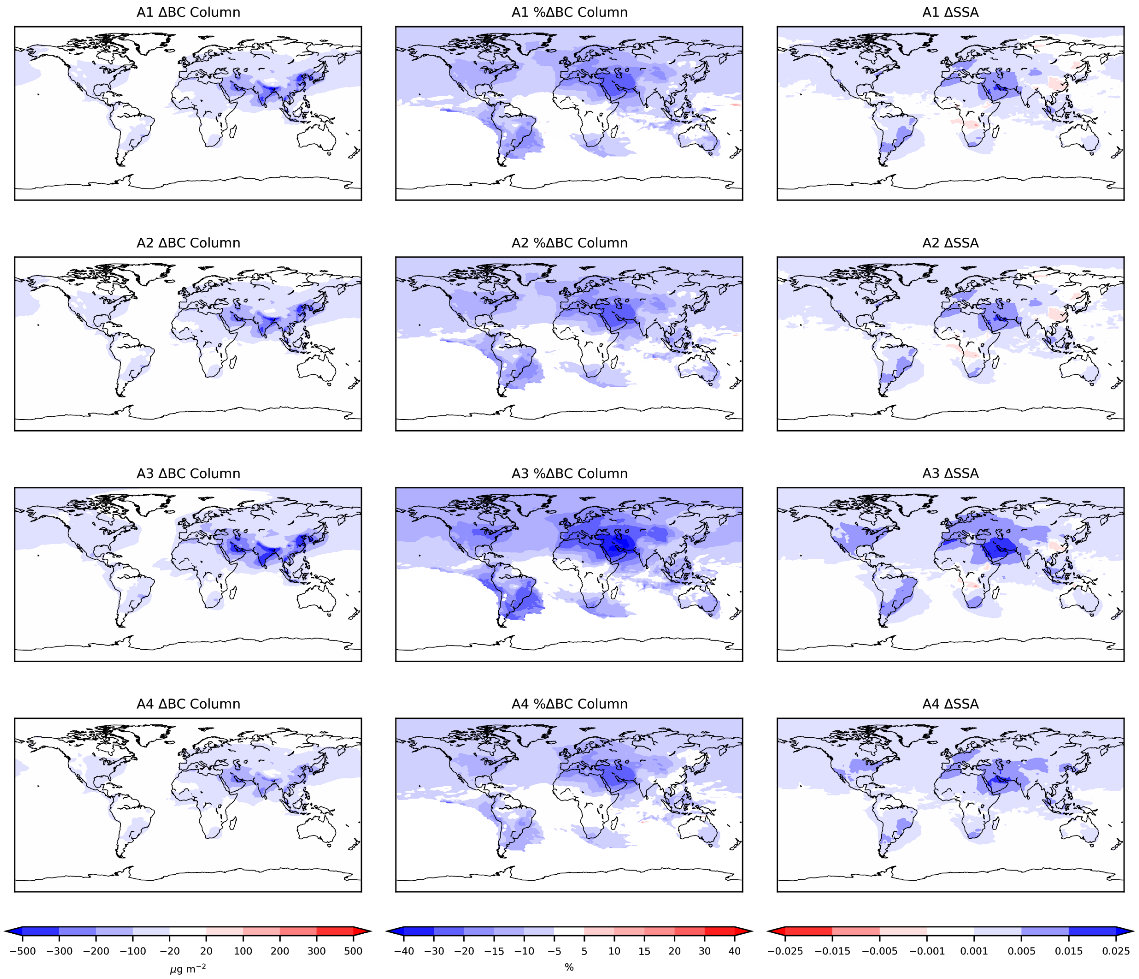

Figure S16. Absolute and percentage changes in black carbon column and change to single-scattering albedo (SSA) (March-May) averaged over three years 2012-2014. Regions with BC columns <10 μ m^{-2} were masked out (control global average column BC column 286 μ m-2).

Text S1: Emission Scenario Description

The emissions scenarios were conceptualised in late March/early April 2020 when verified data concerning the impact of lockdowns on anthropogenic sector emissions was not plentiful or widely available. In order to best estimate the reductions, we compiled information from several sources which are detailed below:

Lockdown measures resulted in an 88% decline in car use in the EU and a 60% decrease in industrial carbon emissions by 25th March (Mallet, 2020), the EEA reported that NO2 concentrations in several cities in southern Europe were around 50% lower than 2019 (European Environment Agency, 2020). In the UK, there was a 60% reduction in all motor vehicle use in the UK (UK Department of Transport, 2020).

International flights from the UK, USA, China, Germany and Japan have decreased 75% from January to the end of March this year (Kommenda, 2020), and european internal flights are estimated to have decreased by 86%. Data from Flightradar (FlightRadar, 2020) was also used to estimate a change in the total global flight by around 50%. Some uncertainty was present early on due to the 'ghost flights' berth requirements law, but the law was later suspended (Morgan, 2020).

The industrial sector was also hit by the COVID-19 lockdowns, but was a lot harder to quantify. It was suggested that EU industrial emissions decreased up to 60% [FT 2020],

Whilst it is likely that many other sectors were affected by the lockdowns, the data at the time provided insufficient evidence to come up with perturbations, so we did not attempt to estimate any of these changes. In this manner, our scenarios most likely represent a lower bound on the actual effect.

Text S2: Methane Concentration Evolution

To estimate the transient change in methane as a result of its lifetime perturbation, a simple kinetic model is considered with an instantaneous 4% increase in methane lifetime. This produces an upper bound estimate for methane concentration as the lifetime change in scenarios A1-A4 are not instantaneous and only one scenario, A3, reaches 4% (Fig. S5). Nevertheless, the results are informative.

In this model, the initial steady state concentration of methane, , is defined in terms of methane flux, , and its lifetime, :

$$[CH_4]_0 = F\tau_0$$

Upon an instantaneous perturbation of methane lifetime to perturbed value, , with an unchanged flux, the methane concentration ceases to be that given by the steady state expression and can be described by the following differential equation:

$$\frac{d[CH_4]}{dt} = F - \frac{1}{\tau}[CH_4]$$

Solving this via separation of variables yields:

$$-\tau\, ln\, |F - \frac{1}{\tau}[CH_4]| = t + c$$

Where c is the constant integration.

Noting that at $t = 0$, $[CH_4] = [CH_4]_0$, the constant of integration, c, can be written as:

$$c = -\tau\, ln\, |F - \frac{1}{\tau}[CH_4]_0|$$

This yields:

$$-\tau\, ln\, |F - \frac{1}{\tau}[CH_4]| = t - \tau\, ln\, |F - \frac{1}{\tau}[CH_4]_0|$$

Dispensing with the moduli and multiplying by $-\tau$yields:

$$ln\,(F - \frac{1}{\tau}[CH_4]) = -\frac{t}{\tau} + ln\,(F - \frac{1}{\tau}[CH_4]_0)$$

$$F - \frac{1}{\tau}[CH_4] = e^{-\frac{t}{\tau}}(F - \frac{1}{\tau}[CH_4]_0)$$

$$\frac{1}{\tau}[CH_4]=F - e^{-\frac{t}{\tau}}(F - \frac{1}{\tau}[CH_4]_0)$$

$$\frac{1}{\tau}[CH_4]=F(1 - e^{-\frac{t}{\tau}}) - \frac{1}{\tau}[CH_4]_0 e^{-\frac{t}{\tau}}$$

$$[CH_4]=F\tau(1 - e^{-\frac{t}{\tau}}) - [CH_4]_0 e^{-\frac{t}{\tau}}$$

Noting that $[CH_4]_0 \ = F\tau_0$

$$[CH_4]=F\tau(1 - e^{-\frac{t}{\tau}}) - F\tau_0 e^{-\frac{t}{\tau}}$$

The ratio, r, of perturbed $[CH_4]$to original $[CH_4]_0$ can be expressed as:

$$r = \frac{[CH_4]}{[CH_4]_0}=\frac{F\tau(1-e^{-\frac{t}{\tau}})-F\tau_0 e^{-\frac{t}{\tau}}}{F\tau_0} = \frac{\tau(1-e^{-\frac{t}{\tau}})}{\tau_0} - e^{-\frac{t}{\tau}}$$

This ratio satisfies the requirements:

At $t = 0$,$r = 1$

As $t \rightarrow 0$, $r \rightarrow \frac{\tau}{\tau_0}$

The ratio depends weakly on the initial methane lifetime but it is clear that several decades are needed for the model to reach a new steady state concentration (Fig. S18). A 4% instantaneous increase in methane lifetime after 3 months, the length of the simulated perturbation, will result in a 0.1% increase in methane concentrations.

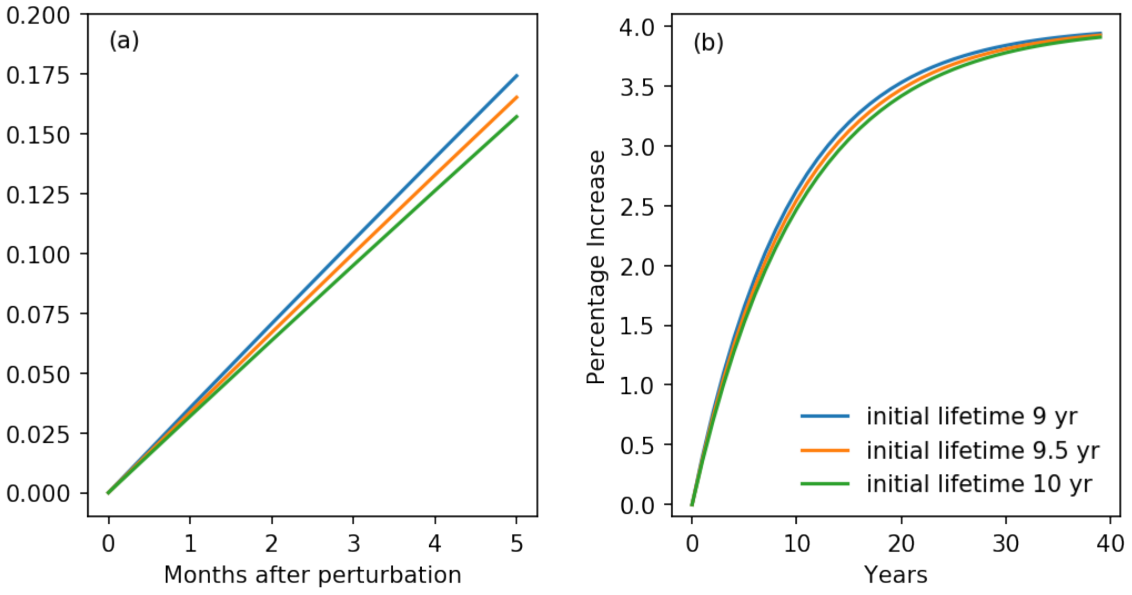

Figure S17. Percentage change in perturbed methane concentration after an instantaneous increase in methane lifetime of 4% using the simple kinetic framework over the first 5 months (b) and 40 years (b). Three initial methane lifetimes were considered.

Table S1. Emitted Species

**Species**
Black carbon
Organic carbon
NO
SO_2
C_2H_6
C_3H_8
HCHO
$(CH_3)_2CO$
CH_3CHO
CH_3OH
Other Organic
NH_3
